# ﻿Taxonomic study on *Sinopoda* Jäger, 1999 (Araneae, Sparassidae, Heteropodinae), with three new species from Korea

**DOI:** 10.3897/zookeys.1114.85493

**Published:** 2022-07-25

**Authors:** Junho Chae, Jun-Gi Lee, Sam-Kyu Kim

**Affiliations:** 1 Department of Biological Sciences, Kangwon National University, Chuncheon 24341, Republic of Korea Kangwon National University Chuncheon Republic of Korea; 2 Applied Biology Program, Division of Bio-Resource Science, College of Agriculture & Life Sciences, Kangwon National University, Chuncheon, 24341, Republic of Korea Kangwon National University Chuncheon Republic of Korea; 3 Interdisciplinary Program in Smart Agriculture, College of Agriculture & Life Sciences, Kangwon National University, Chuncheon, 24341, Republic of Korea Kangwon National University Chuncheon Republic of Korea

**Keywords:** Biodiversity, huntsman spiders, Northeast Asia, revalidation, taxonomy

## Abstract

The genus *Sinopoda* Jäger, 1999 is a group of huntsman spiders (Araneae: Sparassidae: Heteropodinae), and currently seven species have been reported in Korea. In this study, three new species are described from Korea, *Sinopodabigibba***sp. nov.**, *Sinopodabogil***sp. nov.**, and *Sinopodapantherina***sp. nov.**; *Sinopodajirisanensis* Kim & Chae, 2013 is revalidated with neotype designation, and had been formerly synonymized with *Sinopodaforcipata* (Karsch, 1881). Additionally, all previous records of *Sinopodastellatops* Jäger & Ono, 2002 and *S.forcipata* from Korea are deemed misidentifications of *S.jirisanensis* and *S.bogil***sp. nov.**, respectively.

## ﻿Introduction

The genus *Sinopoda* Jäger, 1999 is a group of huntsman spiders (Araneae: Sparassidae), which was designated as a new group comprising some Asian species formerly described under *Heteropoda* Latreille, 1804. To date, 133 species of this genus have been described, of which more than half described in China, while the others are also widely distributed in the east, south, and southeast Asia, ranging from India to Japan (World Spider Catalog 2022).

Currently only seven species of the genus have been recorded from Korea (World Spider Catalog 2022). The first formal study of Korean *Sinopoda* was conducted by Paik (1968), which includes the description of two species, *Sinopodastellata* (Schenkel, 1963) and *Sinopodakoreana* (Paik, 1968) (both under *Heteropoda*; the former was later treated as a misidentification of *Sinopodastellatops* Jäger & Ono, 2002). Later, Kim (2009) reviewed the Korean members, describing *Sinopodaforcipata* (Karsch, 1881) as a previously unrecorded species. The endemic species diversity of Korean *Sinopoda* had been noted by some small studies describing five additional new species before 2015 (Kim et al. 2013, 2014, 2015; Kim and Chae 2013; Kim and Ye 2015), but four of them, viz, *Sinopodaclivus* Kim, Chae & Kim, 2013, *Sinopodajirisanensis* Kim & Chae, 2013, *Sinopodaaureola* Kim, Lee & Lee, 2014, and *Sinopodayeoseodoensis* Kim & Ye, 2015 were synonymized with previously known species in a checklist of Korean spiders (Yoo et al. 2015). However, Lee et al. (2016) noted that the synonymization was not clearly justified since taxonomic notes on their treatment was not provided, and primarily revalidated *S.aureola* and provided descriptions of two new species. According to that study, the other three previously synonymized species could also be removed from their respective senior species. Moreover, a large number of species of the genus have been reported as a result of recent taxonomic works, adding 64 new species to the Asian region ([Bibr B20][Bibr B1]). In this respect, the diversity of Korean *Sinopoda* still remains unexplored, and potential new species await discovery.

The purposes of this study are as follows: 1) describing three new *Sinopoda* species from South Korea; 2) revalidating *S.jirisanensis* as a valid species; 3) correcting misidentifications of Korean records of two previously known species. Detailed descriptions, photographs, and illustrations of four species are provided with taxonomic remarks on these species.

## ﻿Materials and methods

All specimens included in this paper were hand-collected and fixed in 80% ethanol. Photographs of living specimens and all habitus fixed in ethanol were taken with a Nikon D7000 DSLR camera with 105 mm macro lens (Nikon, Tokyo, Japan). Male palps and female genitalia were examined and photographed using an Olympus SZX10 stereomicroscope (Olympus, Tokyo, Japan) and a digital camera (Sony a6000; Sony, Tokyo, Japan) mounted on the microscope after detaching and dissecting the parts from the bodies. Hairs on the ventral surface of cymbium of male palp were removed for accurate observation of embolic division. Epigynes were dissected from opisthosoma and cleared in 10% KOH solution in 75 °C for 20 minutes to examine the internal duct systems. Photographs were taken at different focal depths and stacked using Helicon Focus 7 software (Helicon Soft Ltd., Kharkiv, Ukraine). All measurements are given in millimeters. Specimens were measured under a stereomicroscope (Olympus SZX10) using HK Basic (Koptic, Yongin, Korea) analytical software. Leg and palp measurements are described as total length (femur, patella, tibia, metatarsus, tarsus). Leg spination patterns followed Jäger (2001): the number of spines are listed for each segment as prolateral, dorsal, retrolateral, ventral, and differences of the left and the right leg are given as left/right. Morphological terminology of copulatory organs follows Grall and Jäger (2020). All specimens, including type specimens in this study, are deposited at the Applied Biology Program, Division of Bio-resource Science, Kangwon National University (**KNU**), Chuncheon, Republic of Korea.

Abbreviations used in this work:

**ALE** anterior lateral eyes,

**AME** anterior median eyes,

**AME–ALE** interval of AME and ALE,

**AME–AME** interval of AME and AME,

**ALE–PLE** interval of ALE and PLE,

**AME–PME** interval of AME and PME,

**AW** anterior width of prosoma,

**C** conductor,

**clypeus AME** clypeus height at AME,

**clypeus ALE** clypeus height at ALE,

**dRTA** dorsal retrolateral tibial apophysis,

**E** embolus,

**EA** embolic apophysis,

**EP** epigynal pocket,

**FB** fusion bubble,

**FD** fertilization duct,

**Fe** femur,

**GA** glandular appendage,

**LL** lateral lobes,

**LS** lobal septum,

**MS** membranous sac,

**Mt** metatarsus,

**OL** opisthosoma length,

**OW** opisthosoma width,

**Pa** patella,

**PL** prosoma length,

**PW** prosoma width,

**PLE** posterior lateral eyes,

**PME** posterior median eyes,

**PME–PLE** interval of PME and PLE,

**PME–PME** interval of PME and PME,

**S** spermathecae,

**SP** spermophore,

**ST** subtegulum,

**SS** slit sensillum,

**TE** tegulum,

**Ti** tibia,

**vRTA** ventral retrolateral tibial apophysis.

## ﻿Taxonomic account

### ﻿Family Sparassidae Bertkau, 1872

**Subfamily Heteropodinae Thorell, 1873**

#### ﻿Genus Sinopoda

Taxon classificationAnimaliaAraneaeSparassidae

Jäger, 1999

2571BC93-2A65-55F0-9A22-2C5D6FBD5924

##### Type species.

*Sarotesforcipatus* Karsch, 1881

##### Diagnosis.

This genus is taxonomically close to the genus *Heteropoda* but has the following combinations of characteristics distinct from *Heteropoda* and other sparassid genera: 1) male palp with bifurcated RTA, 2) conductor membranous and arising from distal anterior part of tegulum, 3) embolus typically with embolic apophysis, 4) epigyne with pair of modified rims, and 5) female vulva uncoiled, typically fused along the median line, divided into basal part and head (= glandular appendage), situated laterally from the entrance of internal duct into the spermathecae (Jäger 1999; Liu et al. 2008; Zhang et al. 2015; Grall and Jäger 2020).

##### Ecological notes.

The genus *Sinopoda* is nocturnal and wanders various types of fields such as slope, leaf litter, cave, the forest floor, and on trees with bark (Jäger 1999; Liu et al. 2008; Zhang et al. 2015; Zhong et al. 2019). The female of this genus commonly attaches her egg sac on a flat, wide surface by wrapping it with silks. During the winter season, the *Sinopoda* species hibernates as juveniles or adults. It is generally known that these huntsman spiders are very difficult to collect since very low numbers exist in their habitats and hand-collecting is the only method for collecting them in the fields (Zhong et al. 2019; Zhang et al. 2021).

#### ﻿ Sinopoda bigibba sp. nov.

Taxon classificationAnimaliaAraneaeSparassidae

3CD5895C-30E7-5DD9-B774-0F3CC2450DAC

https://zoobank.org/37C74082-AFB2-4342-AF04-F5651A66C96F

**Figure 1.**
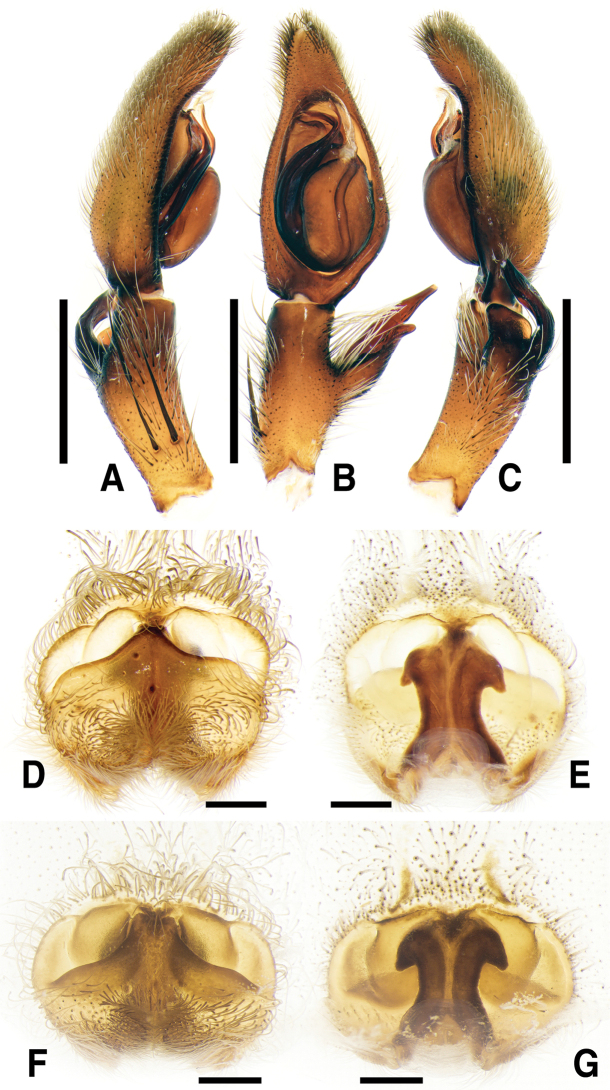
*Sinopodabigibba* sp. nov., male palp and female epigyne **A–C** male palp (**A** prolateral **B** ventral **C** retrolateral) **D, E** female copulatory organ from Is. Gukhwado (**D** ventral **E** dorsal) **F, G** female copulatory organ from Is. Gureopdo (**F** ventral **G** dorsal). Scale bars: 2.0 mm (**A–C**); 0.5 mm (**D–G**).

**Figure 2.**
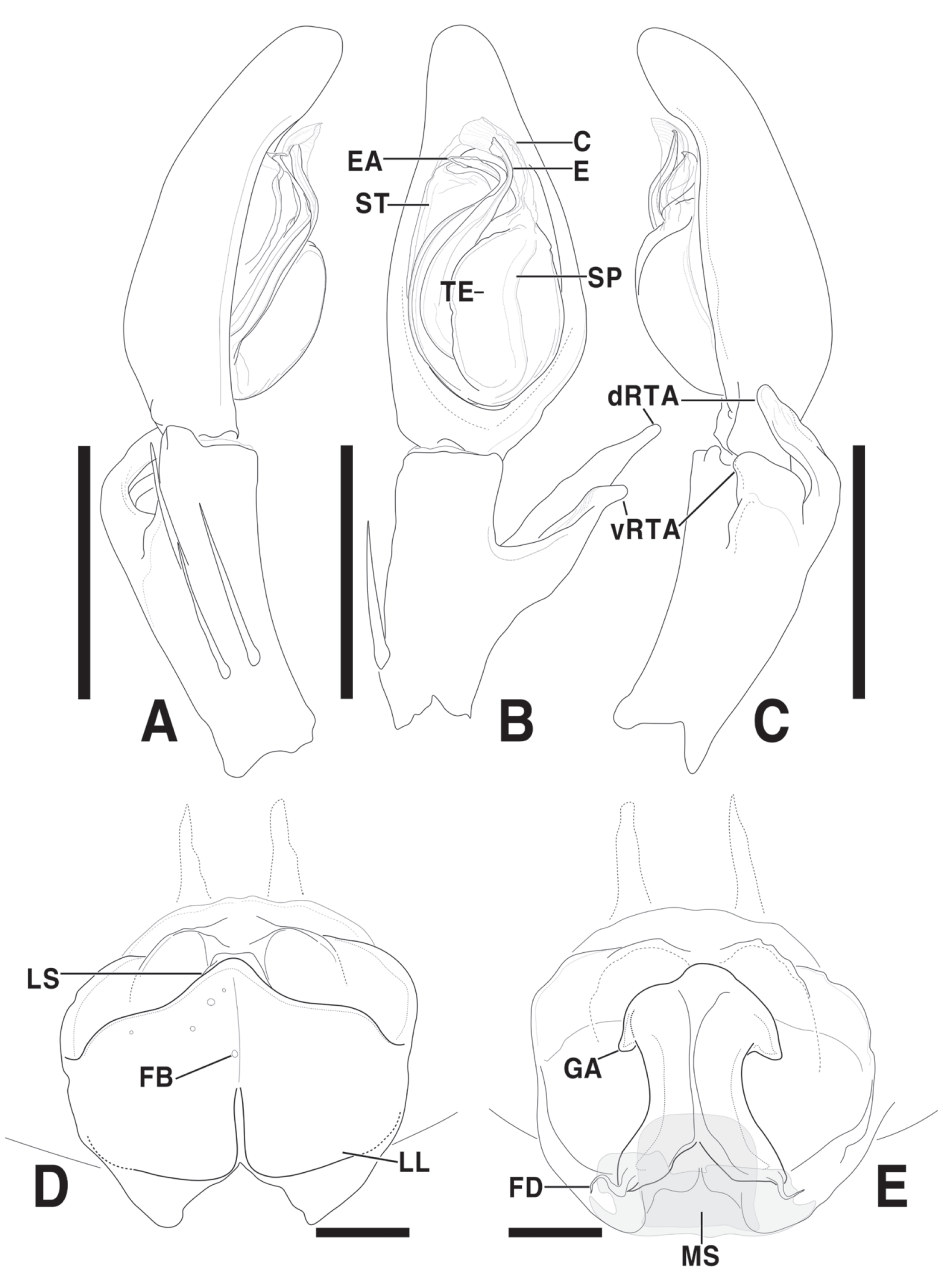
*Sinopodabigibba* sp. nov., illustrations of male palp and female epigyne **A–C** male palp (**A** prolateral **B** ventral **C** retrolateral) **D, E** female copulatory organ from Is. Gukhwado (**D** ventral **E** dorsal). Abbreviations: **C** conductor **dRTA** dorsal branch of retrolateral tibial apophysis **E** embolus **EA** embolic apophysis **FB** fusion bubble **FD** fertilization duct **GA** glandular appendage **LL** lateral lobes **LS** lobal septum **MS** membranous duct **SP** spermophore **ST** subtegulum **TE** tegulum **vRTA** ventral branch of retrolateral tibial apophysis. Scale bars: 2.0 mm (**A–C**); 0.5 mm (**D, E**).

**Figure 3.**
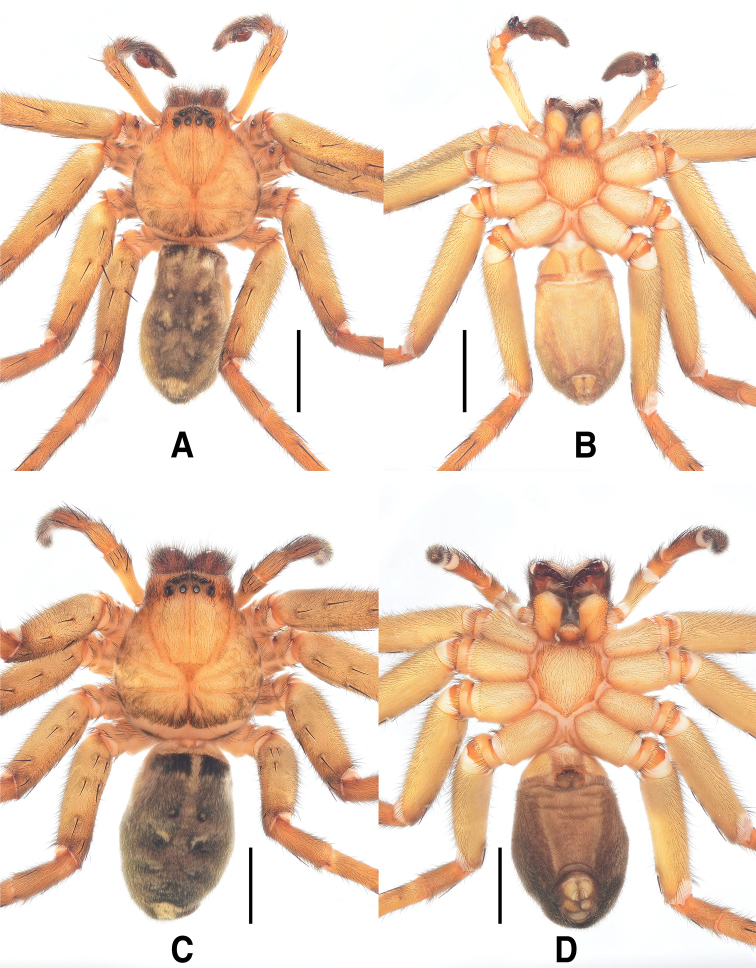
*Sinopodabigibba* sp. nov., habitus in ethanol **A, B** male holotype (**A** dorsal **B** ventral) **C, D** female paratype from Is. Gukhwado (**C** dorsal **D** ventral). Scale bars: 5.0 mm.

Figs 1 , 2 , 3 , 13C

##### Type material.

***Holotype*** ♂ **Republic Of Korea**: Gyeonggi-do, Hwaseong-si, Is. Gukhwado, bottom part of mixed forest; 37°03.50'N, 126°33.22'E; ca. 13 m; 19 Sep. 2019; D. H. Kim leg. ***Paratypes*** 2 ♂♂ 2 ♀♀ same data as holotype. 1 ♀ Chungcheongnam-do, Taean-gun, Wonbuk-myeon, Crack of embankment; 36°51.65'N, 126°11.65'E; ca. 47 m; 17 Jul. 2015; J. Chae leg. 1 ♂ 1 ♀ Incheon, Ongjin-gun, Is. Gureopdo, rock piles of pine tree forest located nearby shoreline; 37°11.33'N, 125°58.88'E; ca. 23 m; 15 Jul. 2020; B. M. Jeong et al. leg. 1 ♀ Jeollabuk-do, Gunsan-si, Is. Yamido, bottom piles of pine tree forest; 35°50.62'N, 126°29.28'E; ca. 20 m; 2 Jun. 2020; J. H. Sohn leg.

##### Etymology.

The specific epithet *bigibba* is a compound word of the prefix *bi*- for two and the Latin adjective *gibbus*, -*a*, -*um* meaning humped, derived from the form of female glandular appendages (Figs 1E, G, 2E).

##### Diagnosis.

This species can be distinguished from other congeners by the combination of following characteristics: Male―embolus with membranous flange extended prolaterally; embolic apophysis tapered distally, with membranous flange slightly extended ventrally; vRTA slightly curved outwardly and distally tapered in ventral view, smooth obtuse-trapezoidal in retrolateral view. Female―posterior muscle sigillae on opisthosoma with pair of large ivory-colored marks; anterolateral margin of lateral lobes sinuous, posterior margin with pair of round humps; lobal septum triangular; glandular appendages very short, slightly protruded.

##### Description.

**Male (*holotype*) Measurements**: Total length: 18.79, PL: 8.47, PW: 7.56, OL: 10.32, OW: 4.32, AW: 4.01. ***Eyes***: AME: 0.36, ALE: 0.53, PME: 0.32, PLE: 0.55, AME–AME: 0.31, AME–ALE: 0.12, PME–PME: 0.42, PME–PLE: 0.43, AME–PME: 0.52, ALE–PLE: 0.56, clypeus AME: 0.29, clypeus ALE: 0.46. Leg formula: 2143, ***Palp***: 12.45 (4.51, 1.95, 2.54, 3.45). ***Legs***: I 43.85 (11.26, 4.18, 11.86, 12.53, 4.02), II 47.48 (12.08, 4.75, 13.05, 13.84, 3.76), III 36.59 (10.51, 3.78, 9.56, 10.04, 2.70), IV 40.04 (10.86, 3.46, 10.47, 11.97, 3.28). Leg formula: II-I-IV-III. **Spination: *Palp***: 131, 101, 2101, 1000. ***Legs***: Fe I, III 323, II 324/323, IV 331/321, Pa I–IV 101, Ti I 1317/1318, II 1118/1218, III–IV 2326, Mt I 1014, II 1014/1013, III 2015/2014, IV 3036. ***Chelicerae***: furrow with three anterior and four posterior teeth.

***Palp***: As per diagnosis (Figs 1A–C, 2A–C). Embolus slender, arising from tegulum at 7:00–7:30-o’clock-position, shorter than embolic apophysis, distally curved. Embolic apophysis wider than embolus, curved perpendicularly. Conductor arising from tegulum at 1-o’clock position. Tegulum slightly covered proximal portion of embolus. Spermophore slightly S-shape. dRTA longer than vRTA, strongly curved nearly perpendicularly and distally tapered. vRTA distinctly wider than dRTA in retrolateral view.

**Coloration in ethanol** (Fig. 3A, B): ***Prosoma***: Carapace yellowish brown, covered with dark brown hairs, lateral margin with dark brown marks, posterior portion lined with dark brown hairs, posterior margin with pale yellow horizontal band, pair of dark brown marks. Cervical groove brown, median groove with brown triangular mark. Sternum pale yellow. ***Opisthosoma***: dorsally covered with grey hairs, anterior portion with pair of black spots laterally and ivory stripe medially, median portion with two pairs of ivory spots near muscle sigillae, posterior portion with two ivory chevron marks and one large ivory triangular mark, ventrally yellowish brown. ***Chelicerae***: reddish brown with dark brown stripes. ***Palp and legs***: yellowish brown and covered with dark brown hairs.

##### Variation.

**Male (*n* = 2) Measurements**: Total length: 13.49–15.13, PL: 7.07–7.90, PW: 6.25–6.91, OL: 6.42–7.23, OW: 3.74–4.28, AW: 3.44–3.69, Leg I: 36.02–38.89.

**Female (*paratype*) Measurements**: Total length: 21.44, PL: 1011, PW: 8.93, OL: 11.33, OW: 7.00, AW: 5.29. ***Eyes***: AME: 0.36, ALE: 0.52, PME: 0.28, PLE: 0.55, AME–AME: 0.43, AME-ALE: 0.25, PME–PME: 0.55, PME–PLE: 0.73, AME–PME: 0.64, ALE–PLE: 0.67, clypeus AME: 0.38, clypeus ALE: 0.44. ***Palp***: 13.10 (4.10, 2.16, 2.67, 4.17). ***Legs***: I 36.05 (10.04, 4.29, 9.53, 9.43, 2.76), II 38.00 (10.81, 4.45, 9.96, 9.85, 2.93), III 32.06 (9.31, 4.03, 8.32, 7.90, 2.50), IV 34.82 (10.01, 3.74, 8.66, 9.85, 2.56). Leg formula: II-I-IV-III. **Spination: *Palp***: 131, 101, 2121, 1014. ***Legs***: Fe I 323/313, II 323, III 323/332, IV 332/321, Pa I–III 101, IV 101/001, Ti I 1018, II 2026, III 2026/2126, IV 2326/2126, Mt I–II 1014, III 2016/2015, IV 3036. ***Chelicerae***: furrow with three anterior and four posterior teeth.

***Copulatory organ***: As per diagnosis (Figs 1D, E, 2D, E). Epigynal field wide as long, with anterior bands, anteromedially with sclerotized epigynal bulges, posteriorly with median indentation. Epigynal pockets running from laterally to anteromedially. Lateral lobes anteriorly fused with fusion bubbles and indistinct median furrow, posteromedially with deep and narrow furrow. Anterolateral margin of lateral lobes sinuous, posterior margin with pair of round humps pointing posteromedially. Lobal septum wide and triangular, anteriorly with slight indentation. Internal duct system longer than wide, anteriorly bulging, posterior part slightly wider than anterior part. Median part of vulva as long as posterior part. Fertilization ducts pointing posterolaterally.

##### Coloration in ethanol.

(Fig. 3C, D): Generally same as male, but coloration darker with more distinct patterns, median portion of opisthosoma with pair of large ivory marks near posterior muscle sigillae.

##### Coloration of live specimen.

(Fig. 13C): ***Prosoma***: Carapace dark brown covered with grey hairs, posterior margin with ivory horizontal band. ***Opisthosoma***: dark grey, laterally paler with many irregular ivory spots, medially with pair of large pale ivory spots.

##### Variation.

**Female (*n* = 4) Measurements**: Total length: 17.33–24.61, PL: 7.29–9.31, PW: 6.02–8.31, OL: 9.48–15.81, OW: 5.40–11.74, AW: 3.69–5.09, Leg I: 23.85–32.27. An intraspecific variation was observed on the width of lobal septum and the presence of median indentation in epigyne (Fig. 1D–G).

##### Distribution.

Republic of Korea (Is. Gukhwado, Is. Gureopdo, Is. Yamido, Taean-gun) (Fig. 15).

##### Remarks.

The male of *Sinopodabigibba* sp. nov. is similar to *Sinopodaaequalis* Zhong, Jäger, Chen & Liu, 2019 (Zhong et al. 2019: 8, figs 4A–C, 5A–D) and *Sinopodabrevis* Zhong, Jäger, Chen & Liu, 2019 (Zhong et al. 2019: 12, figs 10A–C, 11A–D) in having similar RTA. However, the new species can be distinguished from the latter by those following characters: 1) embolus broadened with membranous flange distally (consistently slender in *S.aequalis* and *S.brevis*) and 2) embolic apophysis distally tapered (blunt in *S.aequalis*).

**Figure 4.**
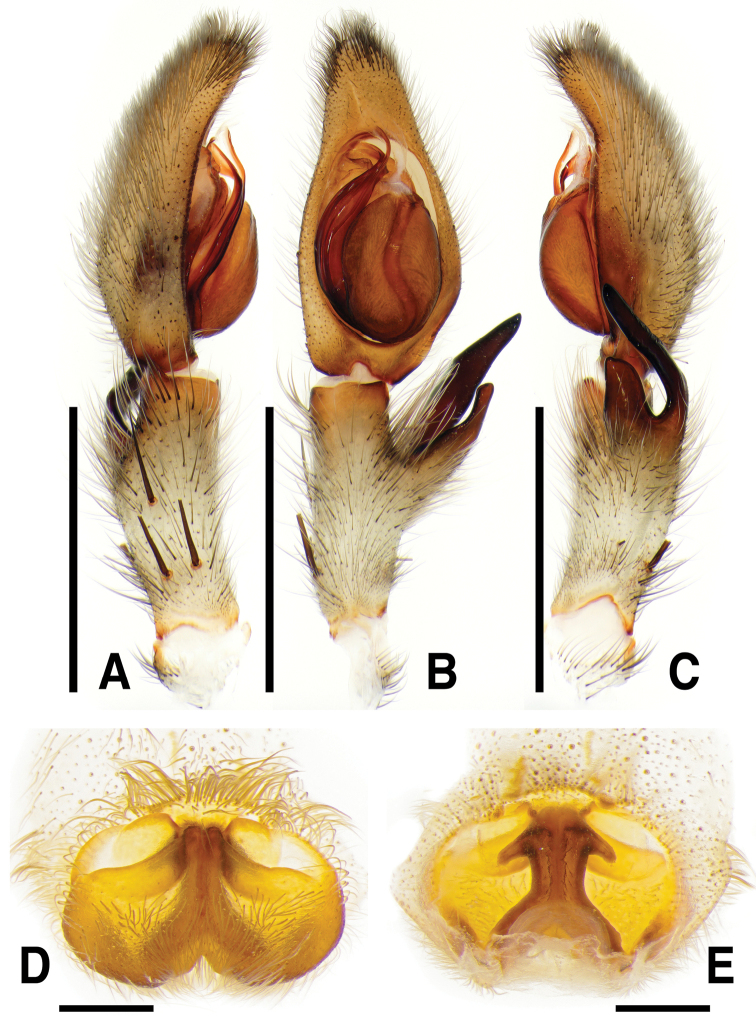
*Sinopodabogil* sp. nov., male palp and female epigyne **A–C** male palp (**A** prolateral **B** ventral **C** retrolateral) **D, E** female copulatory organ (**D** ventral **E** dorsal). Scale bars: 2.0 mm (**A–C**); 0.5 mm (**D, E**).

**Figure 5.**
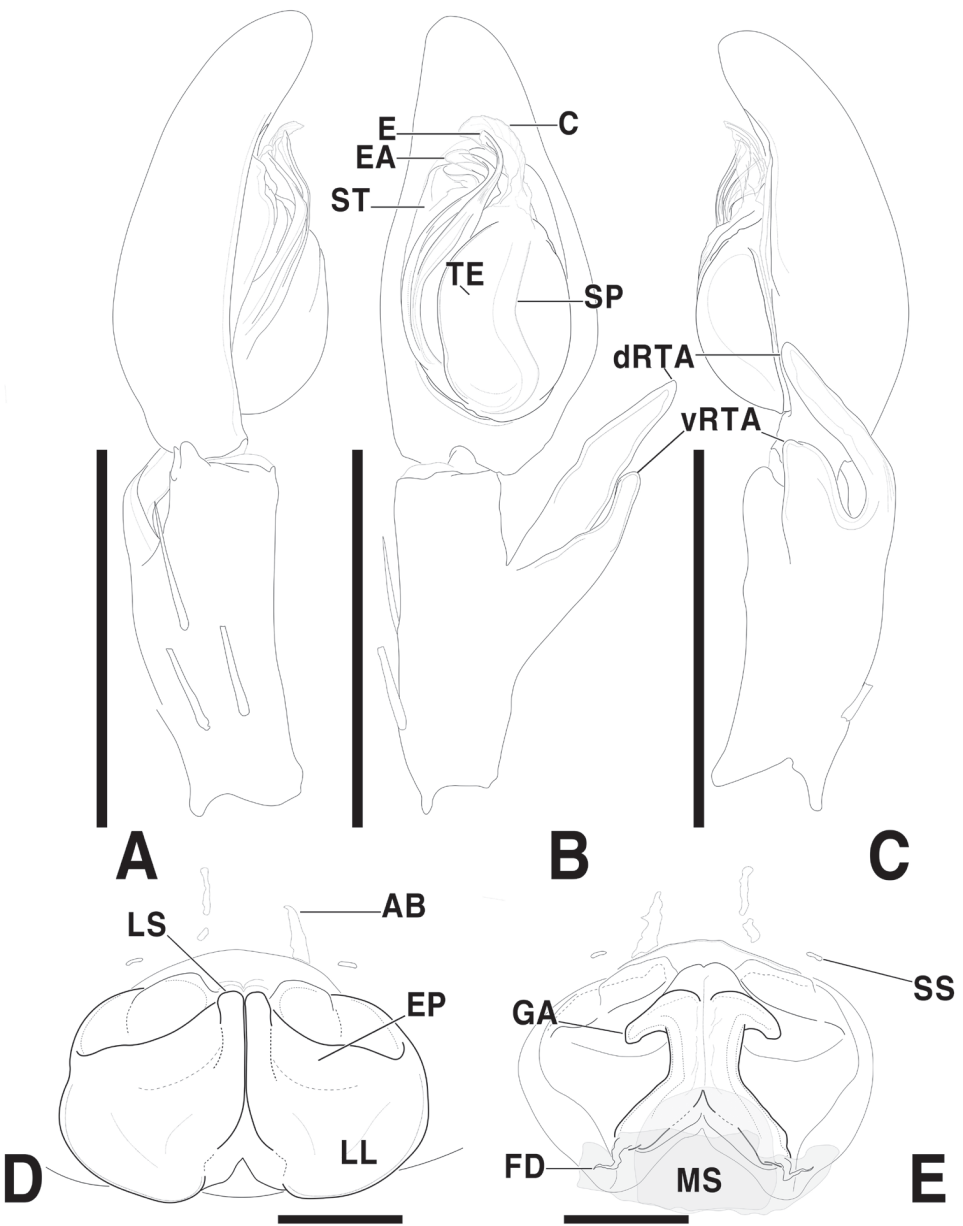
*Sinopodabogil* sp. nov., illustrations of male palp and female epigyne **A–C** male palp (**A** prolateral **B** ventral **C** retrolateral) **D, E** female copulatory organ (**D** ventral **E** dorsal) Abbreviations: **AB** anterior band **C** conductor **dRTA** dorsal branch of retrolateral tibial apophysis **E** embolus **EA** embolic apophysis **EP** epigynal pocket **FD** fertilization duct **GA** glandular appendage **LL** lateral lobes **LS** lobal septum **MS** membranous duct **SP** spermophore **SS** slit sensillum **ST** subtegulum **TE** tegulum **vRTA** ventral branch of retrolateral tibial apophysis. Scale bars: 2.0 mm (**A–C**); 0.5 mm (**D, E**).

The female of the new species is similar to *S.brevis* (Zhong et al. 2019: 12, figs 10D, E, 12A, B) in having distinct sclerotized epigynal bulges and a posterior hump on the lateral lobe, but clearly distinguished by: 1) lobal septum wider (narrower in *S.brevis*), 2) medial indentation vague or absent (distinct in *S.brevis*), and 3) glandular appendages very short and nearly fused with anterior part of internal duct system (well-developed and distinctly separated with anterior part of internal duct system in *S.brevis*).

#### ﻿ Sinopoda bogil sp. nov.

Taxon classificationAnimaliaAraneaeSparassidae

30254AF0-3DC8-5F17-9CFC-9430E234F9CC

https://zoobank.org/C39FA9F6-E90F-4B80-9CE3-9F9980DBBCB8

Figs 4 , 5 , 6

Sinopoda forcipata : Kim, 2009: 238, figs 1A–I, 3A–C (*nec* Karsch, 1881) (misidentification).

##### Type material.

***Holotype*** ♂ **Republic Of Korea**: Jeollanam-do, Wando-gun, Is. Bogildo, leaf litter slope of mixed forest; 34°09.53'N, 126°32.62'E; ca. 176 m; 5 Apr. 2021; D. Y. Song leg. ***Paratype*** 1 ♀ same data as holotype.

##### Etymology.

The specific epithet *bogil* is derived from the type locality, Is. Bogildo; noun.

##### Diagnosis.

This species can be distinguished from other congeners by the combination of following characteristics: Male―embolus with membranous flange extended prolaterally, broadened in distal; embolic apophysis with blunt triangular membranous tip; tegulum pisiform, with slightly convex prolateral portion; vRTA slightly curved inwardly and distally blunt in ventral view, thumb-shaped in retrolateral view. Female―lateral lobes with distinct median furrow, posteromedially concave; anterolateral margin of lateral lobes slightly sinuated, posterior margin with pair of slightly protruded humps; lobal septum triangular with anterior indentation; glandular appendages linear and slightly curved posterolaterally in apex, distinctly shorter than posterior part of vulva.

##### Description.

**Male (*holotype*) Measurements**: Total length: 12.62, PL: 6.09, PW: 5.48, OL: 6.53, OW: 3.54, AW: 3.03. ***Eyes***: AME: 0.25, ALE: 0.42, PME: 0.35, PLE: 0.49, AME–AME: 0.30, AME–ALE: 0.14, PME–PME: 0.34, PME–PLE: 0.49, AME–PME: 0.49, ALE–PLE: 0.51, clypeus AME: 0.26, clypeus ALE: 0.28. ***Palp***: 9.06 (2.94, 1.72, 1.84, 2.56). ***Legs***: I 28.46 (7.50, 2.97, 7.72, 7.65, 2.62), II 31.30 (8.45, 3.40, 7.95, 8.63, 2.87), III 23.99 (7.03, 2.44, 6.28, 6.36, 1.88), IV 26.06 (7.26, 2.55, 6.69, 7.37, 2.19). Leg formula: II-I-IV-III. **Spination: *Palp***: 131, 101, 2111, 1000. ***Legs***: Fe I 323/312, II–III 323, IV 331, Pa I 101/001, II–IV 101, Ti I 1418/1116, II 1418, III 2326, IV 3236, Mt I 2120/2202, II 2024, III 3034, IV 3036. ***Chelicerae***: furrow with three anterior and four posterior teeth.

***Palp***: As per diagnosis (Figs 4A–C, 5A–C). Embolus slender, arising from tegulum at 7:30–8-o’clock-position, slightly shorter than embolic apophysis, distally curved. Embolic apophysis wider than embolus. Conductor arising from tegulum at 12:30-o’clock-position. Tegulum slightly covered proximal portion of embolus. Spermophore slightly S-shape. dRTA longer than vRTA, proximally strongly curved, distally tapered. vRTA wider than dRTA in retrolateral view.

**Figure 6.**
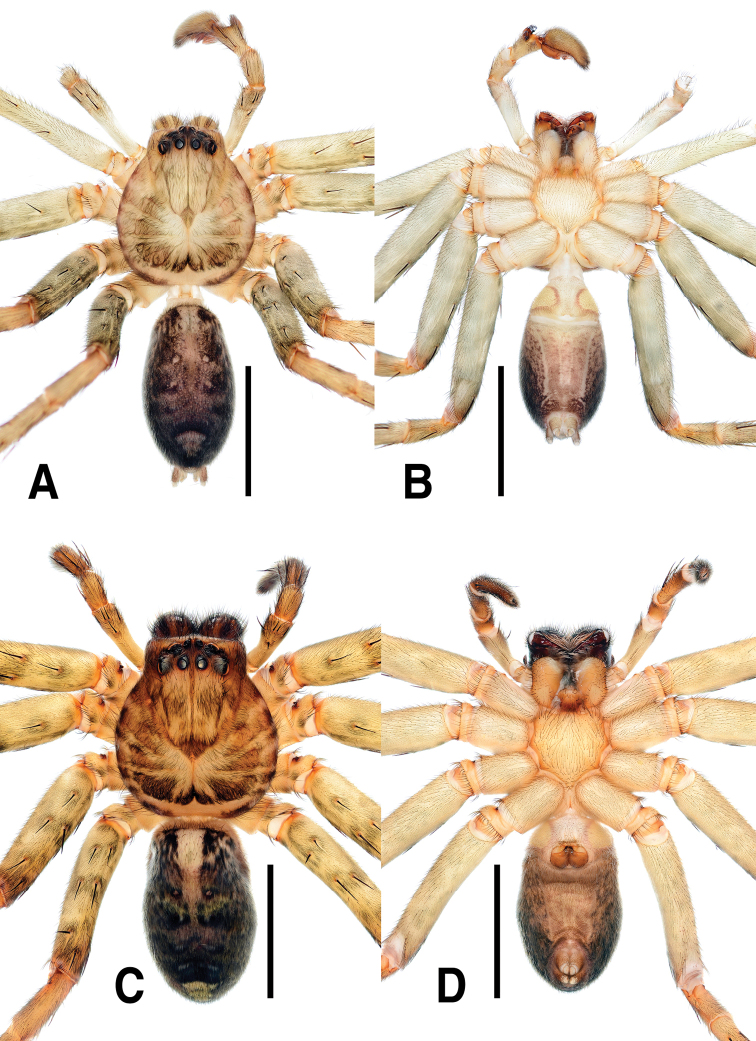
*Sinopodabogil* sp. nov., habitus in ethanol **A, B** male holotype (**A** dorsal **B** ventral) **C, D** female paratype (**C** dorsal **D** ventral). Scale bars: 5.0 mm.

**Coloration in ethanol.** (Fig. 6A, B): ***Prosoma***: Carapace ivory, anteriorly and medially with dark khaki-green hairs making radial pattern, lateral and posterolateral margin with many reddish brown marks, posterior margin with pale yellow horizontal band. Cervical groove yellowish brown and median groove with yellowish brown triangular mark covered with dark hairs. Sternum ivory. ***Opisthosoma***: dorsally covered with khaki brown hairs, anterior portion with many pairs of irregular black spots laterally and longitudinal ivory stripe medially, median portion with brown laciniate longitudinal pattern medially, lateral portion with irregular dark brown marks, posterior portion with large ivory triangular mark, ventrally brown medially, laterally with dark brown spots. ***Chelicerae***: yellowish brown with two brown stripes. ***Palp and legs***: femur pale green covered with dark grey hairs, elsewhere yellowish brown.

**Coloration of live specimen.** (Fig. 13A): ***Prosoma***: Carapace covered with dark grey hairs and thoracic area with pale ivory radial stripe medially. ***Opisthosoma***: laterally with black irregular marks, medially with ivory laciniate longitudinal stripe, posteriorly with two ivory chevron and triangular mark. ***Palp and legs***: femur with dark grey hairs, elsewhere yellowish brown.

**Female (*paratype*) Measurements**: Total length: 13.22, PL: 6.69, PW: 5.72, OL: 6.53, OW: 4.09, AW: 3.74. ***Eyes***: AME: 0.26, ALE: 0.49, PME: 0.36, PLE: 0.45, AME–AME: 0.27, AME–ALE: 0.12, PME–PME: 0.43, PME–PLE: 0.56, AME–PME: 0.55, ALE–PLE: 0.58, clypeus AME: 0.38, clypeus ALE: 0.36. ***Palp***: 9.59 (2.98, 1.44, 2.33, 2.84). ***Legs***: I 23.48 (6.76, 2.69, 6.20, 5.74, 2.09), II 25.98 (7.73, 2.91, 6.69, 6.40, 2.25), III 22.29 (6.71, 2.92, 5.73, 5.29, 1.64), IV 24.41 (6.82, 2.49, 6.23, 6.74, 2.13). Leg formula: II-IV-I-III. **Spination: *Palp***: 131, 101, 2121, 1014. ***Legs***: Fe I–III 323, IV 331, Pa I, IV 001, II–III 101, Ti I–II 1018, III 2126, IV 2228/2328, Mt I 0014, II 1014, III 2016, IV 3036. ***Chelicerae***: furrow with three anterior and four posterior teeth.

***Copulatory organ***: As per diagnosis (Figs 4D, E, 5D, E). Epigynal field wider than long, with two anterior bands and slit sensilla, anteromedially with sclerotized epigynal bulges. Epigynal pockets running from laterally to anteromedially. Internal duct system longer than wide, fused along median line, anteriorly bulging, posterior part much wider than anterior part. Median part of vulva shorter than posterior part. Fertilization ducts curved and pointing posterolaterally.

**Coloration in ethanol.** (Fig. 6C, D): Generally same as male, but coloration darker and more yellowish, with more distinct patterns. Leg spines with dark brown ring patterns.

**Coloration of live specimen.** (Fig. 13B): Generally same as male, but coloration yellowish. Median laciniate pattern on opisthosoma indistinct, muscle sigillae with black round spots.

##### Distribution.

Republic of Korea (known only from the type locality) (Fig. 15).

##### Remarks.

*Sinopodabogil* sp. nov. has been described as *Sinopodaforcipata* (Karsch, 1881) (Jäger 1999: 20, figs 1–4, 6, 7; Jäger and Ono 2000: 52, figs 27–34) in Korea (Kim 2009: 238, figs 1A–I, 3A–C), however this species can be readily distinguished by the following characteristics: 1) angled portion of embolic apophysis with retrolateral protrusion (without protrusion in *S.forcipata*), 2) male tegulum pisiform, with slightly convex prolateral portion (droplet shaped, with strongly convex prolateral portion in *S.forcipata*), 3) vRTA slightly sinuated, twice as wide as dRTA, with apex pointing toward cymbium in retrolateral view (distinctly sinuated, less than twice as wide as dRTA, with apex pointing ventrally in retrolateral view in *S.forcipata*), 4) medial indentation of epigyne extending from anterior part of lobal septum to posterior part of epigynal field (medial indentation extending from median to posterior part of epigynal field in *S.forcipata*), 5) epigynal bulges present (absent in *S.forcipata*), and 6) distal portion of posterior part of internal duct system linear (strongly swollen and pointing laterally in *S.forcipata*).

The most comparable species with male of *Sinopodabogil* sp. nov. is *Sinopodabigibba* sp. nov. (Figs 1A–C, 2A–C) in having broadened embolus tip and similar RTA structure in retrolateral view, but *S.bogil* sp. nov. can be distinguished by following characteristics: 1) embolic apophysis distally blunt (distally tapered in *S.bigibba* sp. nov.), 2) vRTA curved inwardly in ventral view, thumb-shaped and distally tapered in retrolateral view (vRTA curved outwardly in ventral view, smooth trapezoidal and distally blunt in retrolateral view in *S.bigibba* sp. nov.).

The female of *Sinopodabogil* sp. nov. is also similar to *Sinopodabigibba* sp. nov. (Figs 1D–G, 2D, E) in having distinct epigynal bulges and short glandular appendages, however this species can be distinguished by following characteristics: 1) lobal septum with anteromedial indentation (without indentation in *S.bigibba*), 2) posterior part of epigynal field slightly protruded (posterior part of epigynal field distinctly protruded posteriorly in *S.bigibba*), and 3) anterior part of internal duct as wide as median part (distinctly wider than median part in *S.bigibba*).

#### ﻿ Sinopoda jirisanensis

Taxon classificationAnimaliaAraneaeSparassidae

Kim & Chae, 2013

312EE598-D86A-5EB5-A4C7-503C4BDBDCB1

Figs 7 , 8 , 9

Sinopoda jirisanensis Kim & Chae, 2013: 184, figs 1–11 (type locality: Korea).
Heteropoda stellata : Paik, 1968: 171, figs 3, 4, 22–29; 1978: 396, fig. 178.1–4 (*nec* Schenkel, 1963) (misidentification).
Sinopoda stellata : Namkung, 2002: 498, fig. 40.2a, b; 2003: 501, fig. 40.2a, b (*nec* Schenkel, 1963) (misidentification).
Sinopoda stellatops : Kim, 2009: 242, figs 2A–D, 3D–F; Kim and Lee 2017: 59, fig. 33A–D (*nec* Jäger & Ono, 2002) (misidentification).
Sinopoda forcipata : Yoo et al. 2015: 78 (synonymization).

##### Type material.

***Holotype*** ♂ **Republic Of Korea**: Jeollabuk-do, Namwon-si, Sannae-myeon; 35°23.14'N, 127°35.05'E; 20 Jun. 2013; J. Chae leg (lost). ***Neotype*** ♂ Jeollabuk-do, Namwon-si, Sannae-myeon, Mt. Jirisan, leaf litter slope of mixed forest; 35°23.11'N, 127°35.08'E; ca. 501 m; 2 Jun. 2020; J. Chae leg. ***Paraneotypes*** 1 ♀ same data as neotype. 1 ♂ 1 ♀ same data as neotype except collecting data: 13 Aug. 2013. 1 ♂ 1 ♀ Jeollabuk-do, Jinan-gun, Jucheon-myeon, leaf litter slope of mixed forest; 35°58.50'N, 127°24.95'E; ca. 298 m; 20 May. 2020; J. H. Sohn leg. 2 ♂♂ 1 ♀ Chungcheongbuk-do, Danyang-gun, Mt. Sobaeksan, mixed forest; 36°57.56'N, 128°25.88'E; ca. 518 m; 30 Apr. 2021; J. G. Lee & J. H. Lee leg. 4 ♂♂ 1 ♀ Gyeongsangbuk-do, Bonghwa-gun, Mt. Gakhwasan, bottom of mixed forest; 36°59.48'N, 128°54.35'E; ca. 725 m; 8 Jul. 2019; J. G. Lee leg. 1 ♀ Gangwon-do, Pyeongchang-gun, Mt. Odaesan, mixed forest nearby wooded valley; 37°44.51'N, 128°35.05'E; ca. 682 m; 18 Sep. 2020; S. K. Kim leg.

##### Diagnosis.

This species can be distinguished from other congeners by the combination of following characteristics: Male―embolus without membranous flange; embolic apophysis distally with ventrally folded membranous extension; tegulum oval, slightly covered proximal portion of embolus; spermophore slightly curved; dRTA finger-like and slightly curved; vRTA blunt triangular. Female―lateral lobes anteriorly fused without median furrow, posteromedially with indentation; anterolateral margin of lateral lobes slightly concave, posterior margin without humps; lobal septum ~ 1/5 as wide as epigynal field width, anteriorly blunt; posterior part of vulva laterally elongated, round; glandular appendages thick, linear, and distally blunt, as long as posterior part of vulva.

##### Description.

**Male (*neotype*) Measurements**: Total length: 12.46, PL: 6.18, PW: 5.60, OL: 6.28, OW: 3.52, AW: 2.75. ***Eyes***: AME: 0.22, ALE: 0.37, PME: 0.28, PLE: 0.40, AME–AME: 0.22, AME–ALE: 0.10, PME–PME: 0.27, PME–PLE: 0.46, AME–PME: 0.41, ALE–PLE: 0.36, clypeus AME: 0.20, clypeus ALE: 0.23. ***Palp***: 9.89 (3.20, 1.49, 2.20, 3.00). ***Legs***: I 32.12 (8.19, 3.09, 8.67, 9.59, 2.58), II 36.31 (9.59, 3.48, 9.78, 10.94, 2.52), III 26.14 (7.21, 2.66, 6.95, 7.32, 2.00), IV 29.51 (7.94, 2.84, 7.57, 8.79, 2.37). Leg formula: II-I-IV-III. **Spination: *Palp***: 131, 101, 2011, 1000. ***Legs***: Fe I–II 323, III 322/323, IV 321; Pa I 101/001, II–III 101, IV 001, Ti I–II 1318, III 2326, IV 2226/2326, Mt I 1014/1013, II 1014, III–IV 3036. ***Chelicerae***: furrow with three anterior and four posterior teeth.

**Figure 7.**
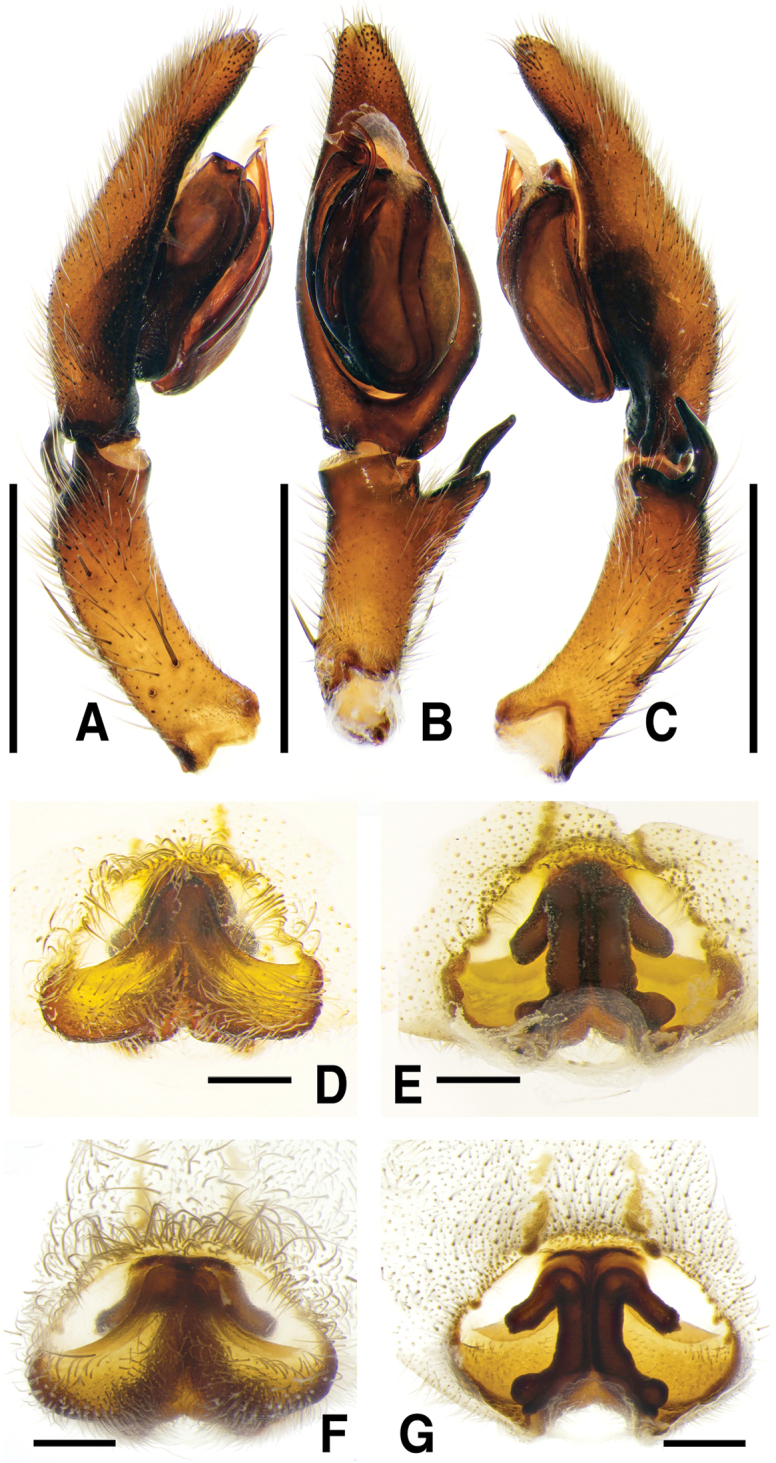
*Sinopodajirisanensis* Kim & Chae, 2013, male palp and female epigyne **A–C** male palp (**A** prolateral **B** ventral **C** retrolateral) **D, E** female copulatory organ from Mt. Jirisan (**D** ventral **E** dorsal) **F, G** female copulatory organ from Jinan-gun (**F** ventral **G** dorsal). Scale bars: 2.0 mm (**A–C**); 0.5 mm (**D–G**).

**Figure 8.**
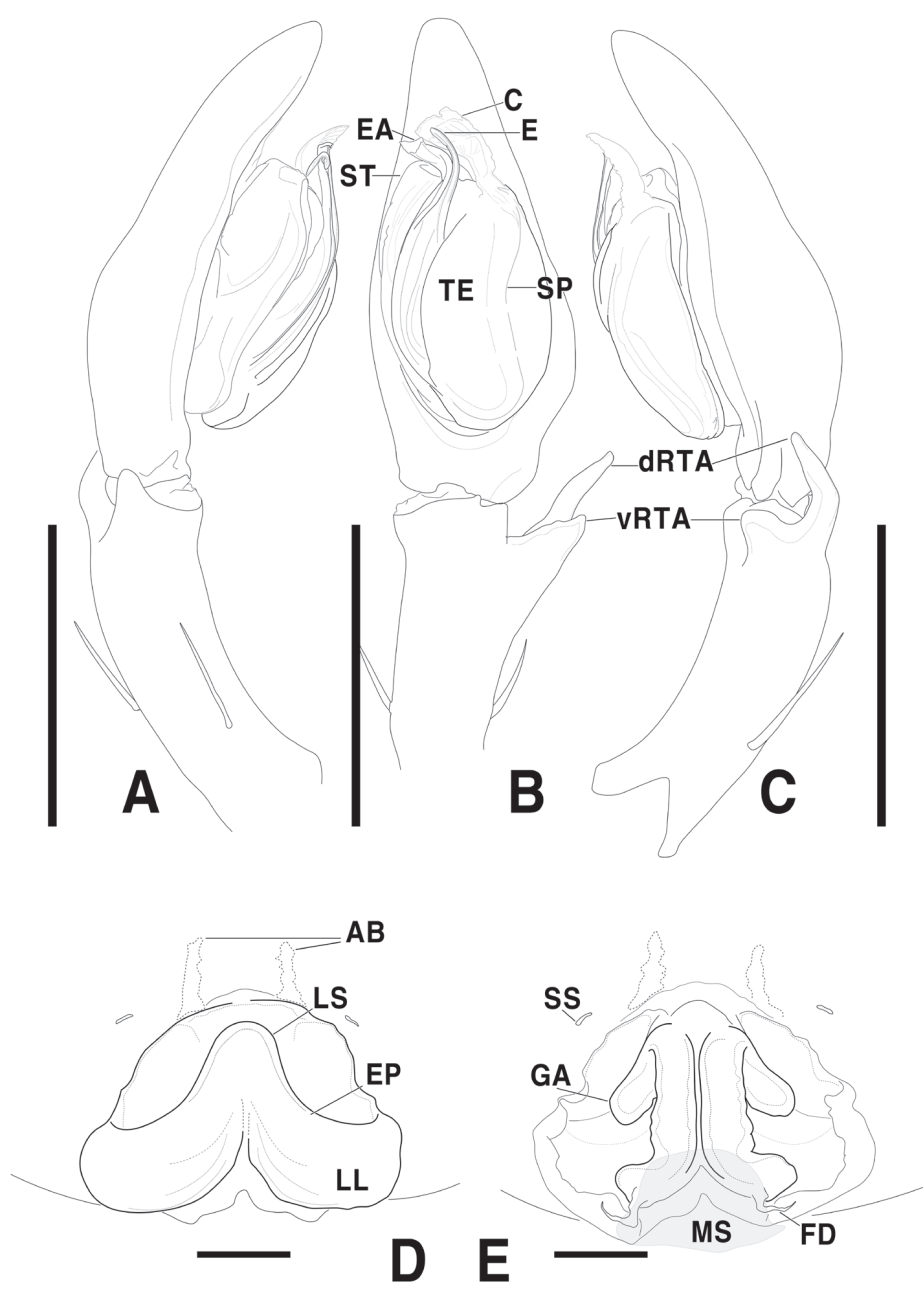
*Sinopodajirisanensis* Kim & Chae, 2013, illustrations of male palp and female epigyne **A–C** male palp (**A** prolateral **B** ventral **C** retrolateral) **D, E** female copulatory organ from Mt. Jirisan (**D** ventral **E** dorsal). Abbreviations: **AB** anterior band **C** conductor **dRTA** dorsal branch of retrolateral tibial apophysis **E** embolus **EA** embolic apophysis **EP** epigynal pocket **FD** fertilization duct **GA** glandular appendage **LL** lateral lobes **LS** lobal septum **MS** membranous duct **SP** spermophore **SS** slit sensillum **ST** subtegulum **TE** tegulum **vRTA** ventral branch of retrolateral tibial apophysis. Scale bars: 2.0 mm (**A–C**); 0.5 mm (**D, E**).

***Palp***: As per diagnosis (Figs 7A–C, 8A–C). Embolus slender, arising from tegulum at 6:30–7-o’clock-position, approximately long as embolic apophysis, distally slightly curved Embolic apophysis wider than embolus, curved perpendicularly. Conductor arising from tegulum at 12:00–12:30-o’ clock-position. Spermophore slightly curved in ventral view. dRTA longer than vRTA and curved outwardly in ventral view. vRTA wider than dRTA.

**Figure 9.**
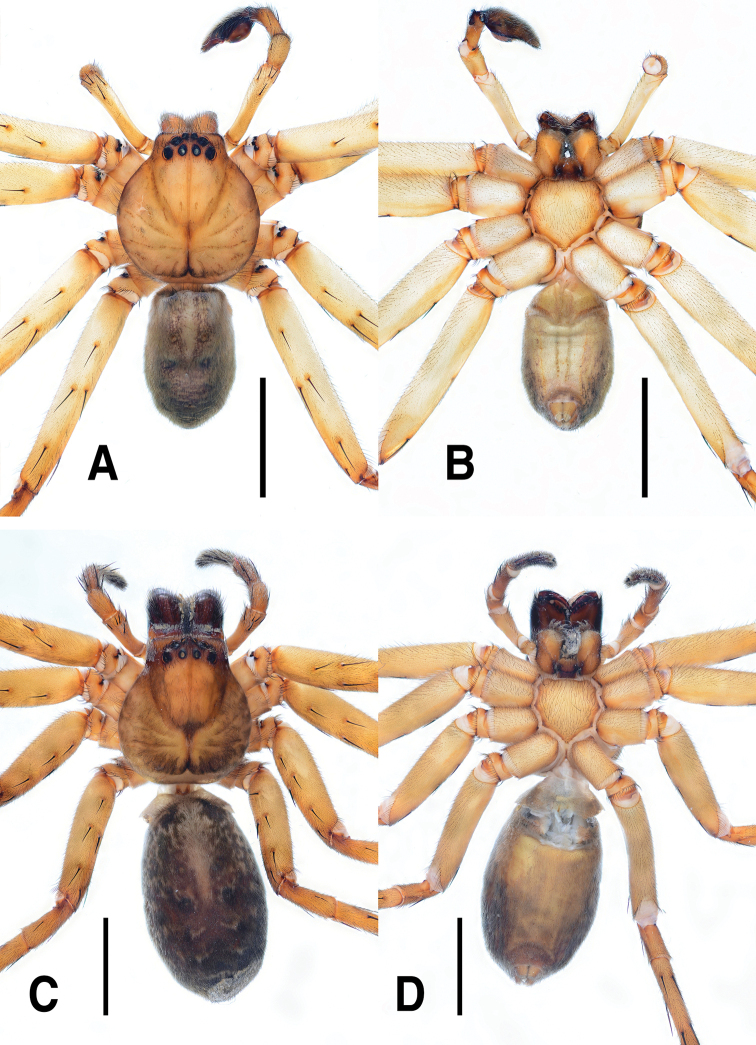
*Sinopodajirisanensis* Kim & Chae, 2013, habitus in ethanol **A, B** male neotype (**A** dorsal **B** ventral) **C, D** female paraneotype from Mt. Jirisan (**C** dorsal **D** ventral). Scale bars: 5.0 mm.

**Coloration in ethanol.** (Fig. 9A, B): Carapace yellowish brown, lateral margin with dark brown marks, posterior portion lined with dark brown hairs, posterior margin with pale yellow horizontal band. Cervical groove, radial groove, median groove distinct, dark brown. Sternum pale yellow. ***Opisthosoma***: dorsally covered with khaki brown hairs, anterior portion with pair of black irregular marks laterally and yellow longitudinal stripe medially, median portion with black spots on muscle sigillae, ventrally pale khaki, with two dark green longitudinal stripes. ***Chelicerae***: reddish brown. ***Palp and legs***: pale yellow.

**Coloration of live specimen.** (Fig. 14A, E): ***Prosoma***: Carapace pale brown, medially with yellow hairs and dark brown hairs making radial pattern on thoracic area. ***Opisthosoma***: khaki brown, anteromedially with yellow hairs, medially with four black spots on muscle sigillae. ***Palp and legs***: yellowish brown, spine with dark brown ring pattern.

**Variation. Male (*n* = 10) Measurements**: Total length: 10.50–14.90, PL: 5.53–7.14, PW: 4.86–5.99, OL: 4.97–7.76, OW: 2.97–4.96, AW: 2.60–3.33, Leg I: 27.63–33.35.

**Female (*paraneotype*) Measurements**: Total length: 15.85, PL: 6.59, PW: 5.63, OL: 9.26, OW: 6.73, AW: 3.53. ***Eyes***: AME: 0.26, ALE: 0.39, PME: 0.27, PLE: 0.36, AME–AME: 0.29, AME–ALE: 0.19, PME–PME: 0.40, PME–PLE: 0.52, AME–PME: 0.45, ALE–PLE: 0.48, clypeus AME: 0.28, clypeus ALE: 0.29. ***Palp***: 8.28 (2.46, 1.42, 1.83, 2.57). ***Legs***: I 21.52 (6.07, 2.66, 5.50, 5.45, 1.84), II 23.83 (6.88, 2.90, 6.15, 5.89, 2.01), III 19.65 (5.84, 2.48, 5.03, 4.78, 1.52), IV 21.63 (5.90, 2.33, 5.39, 6.03, 1.98). Leg formula: II-IV-I-III. **Spination: *Palp***: 131, 101, 2121, 1014. ***Legs***: Fe I–III 323, IV 321, Pa I–IV 101, Ti I 1218/1018, II 1118/1218, III 2226, IV 2326, Mt I–II 1014, III 2326, IV 3036. ***Chelicerae***: furrow with three anterior and four posterior teeth.

***Copulatory organ***: As per diagnosis (Figs 7D, E, 8D, E). Epigynal field wide as long, with two anterior bands and slit sensilla, posteriorly with median indentation. Epigynal pockets running from posterolaterally to anteromedially. Internal duct system long as wide, anteriorly slightly bulging, posterior part wide as anterior part, laterally elongated roundly. Median part of vulva longer than posterior part. Fertilization ducts pointing posterolaterally.

**Coloration in ethanol.** (Fig. 9C, D): Generally similar to male, but with dark coloration and distinct patterns. Carapace with dark brown hairs making radial pattern on thoracic area. Opisthosoma dark brown, dorsally with longitudinal ivory stripe anteromedially, black round spots on muscle sigillae, two ivory-colored chevrons and thin horizontal mark posteromedially, laterally with small irregular ivory marks.

**Coloration of live specimen.** (Fig. 14B–D): Generally similar to male, but with reddish coloration and distinct patterns. Lateral margin of carapace with dark brown marks. Opisthosoma dark grey, dorsally with longitudinal ivory stripe anteromedially, black round spots on muscle sigillae, two ivory chevrons and thin horizontal mark posteromedially, laterally with small irregular ivory marks.

**Variation. Female (*n* = 7) Measurements**: Total length: 10.50–14.90, PL: 5.53–7.14, PW: 4.86–5.99, OL: 4.97–7.76, OW: 2.97–4.96, Leg I: 27.63–33.35.

In some specimens, some variations are observed in which the internal duct system is wide as long without medial protuberance and the posterior part is distinctly curved toward the dorsal direction. Intraspecific variations were observed in the shape of lateral lobes and the width of lobal septum (Fig. 7F, G).

##### Distribution.

Republic of Korea (Mt. Jirisan, Mt. Sobaeksan, Jinan-gun, Mt. Gakhwasan, and Mt. Odaesan) (Fig. 15).

##### Remarks.

It is known that the holotype and paratype were lost due to fire in the depository (JooPil Spider Museum, Namyangju-si, Korea). For taxonomic stability of the species, we designate the neotype and paraneotypes of this species. Some of these materials were collected from the same locality as holotype.

*Sinopodajirisanensis* Kim & Chae, 2013 was synonymized to *Sinopodaforcipata* (Karsch, 1881) (Yoo et al. 2015: 6), however *S.jirisanensis* can be easily distinguished from *S.forcipata* (Jäger 1999: 20, figs 1–4, 6–7; Jäger and Ono 2000: 52, figs 27–34) by the following characteristics: 1) embolus consistently slender, without membranous flange (distally broadened with membranous flange in *S.forcipata*), 2) distal portion of embolic apophysis distinctly longer than anterior width of subtegulum, with folded extension (slightly shorter than anterior width of subtegulum, without folded extension in *S.forcipata*), 3) dRTA distinctly shorter than tibia (as long as tibia in *S.forcipata*), 4) lobal septum ~ 1/5 of epigynal field width (~ 1/9 in *S.forcipata*), and 5) posteromedial portion of lateral lobes without humps (with pair of round humps pointing posteromedially in *S.forcipata*). Judging from diagnostic characteristics mentioned above, this species should be removed from a junior synonym of *S.forcipata* and revalidated as a good species.

Moreover, this species has been erroneously described as *Sinopodastellatops* Jäger & Ono, 2002 (Jäger and Ono 2002: 119–121, figs 42–64) in Korea (Paik 1968: 171, figs 3, 4, 22–29; Kim 2009: figs 2A–D, 3D–F) for many years. All former descriptions and records on *S.stellatops* from Korea were misidentifications of *S.jirisanensis* which can be easily distinguished from *S.stellatops* by the following characteristics: 1) embolus and embolic apophysis slightly curved (both convulsively bent in *S.stellatops*), 2) tegulum oval (droplet-shaped in *S.stellatops*), 3) dRTA ~ 3 × as long as vRTA, slightly curved and arising distally from tibia (dRTA less than twice as long as vRTA, strongly curved and arising medial tibia in *S.stellatops*), 4) vRTA short and distally blunt, arising from distal tibia (vRTA finger-like, arising from medial tibia in *S.stellatops*), 5) posteromedial field of epigyne distinctly concave with indentation along lateral lobes (slightly concave without indentation in *S.stellatops*), and 6) glandular appendage as long as posterior part of internal duct system (much longer than posterior part of internal duct system in *S.stellatops*).

The male of this species resembles *Sinopodaogatai* Jäger & Ono, 2002 (Jäger and Ono 2002: 118, figs 37–41) in having embolus without membranous flange and short RTA structures, but it can be distinguished from the latter by: 1) embolic apophysis with folded distal extension (without folded extension in *S.ogatai*), 2) prolateral margin of tegulum not strongly extended (strongly extended and covered proximal portion of embolus in *S.ogatai*), and 3) vRTA triangular in ventral view (finger-like in ventral view in *S.ogatai*).

#### ﻿ Sinopoda pantherina sp. nov.

Taxon classificationAnimaliaAraneaeSparassidae

4BD78F2C-3640-55A9-9807-1583E55B6E21

https://zoobank.org/927F374F-4C25-4B86-83F0-915459FB171D

**Figure 10.**
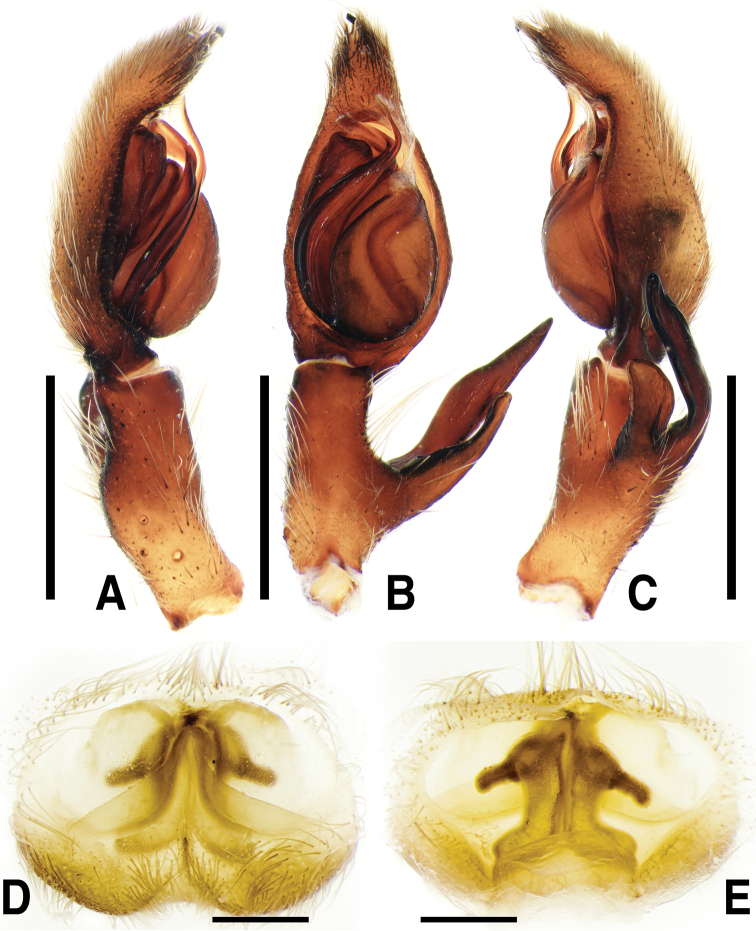
*Sinopodapantherina* sp. nov., male palp and female epigyne **A–C** male palp (**A** prolateral **B** ventral **C** retrolateral) **D, E** female copulatory organ (**D** ventral **E** dorsal). Scale bars: 2.0 mm (**A–C**); 0.5 mm (**D, E**).

**Figure 11.**
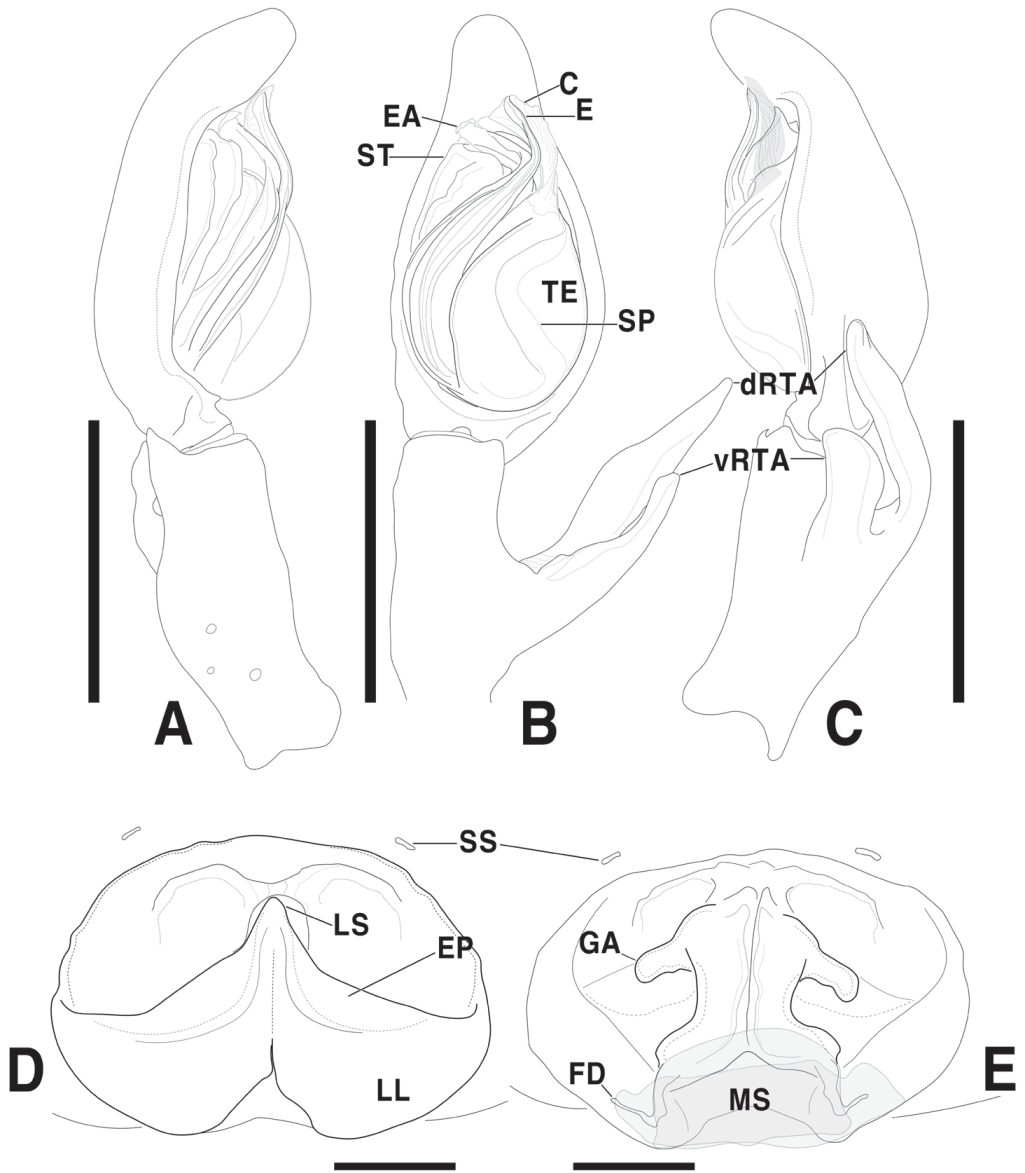
*Sinopodapantherina* sp. nov., illustrations of male palp and female epigyne **A–C** male palp (**A** prolateral **B** ventral **C** retrolateral) **D, E** female copulatory organ (**D** ventral **E** dorsal). Abbreviations: **C** conductor **dRTA** dorsal branch of retrolateral tibial apophysis **E** embolus **EA** embolic apophysis **EP** epigynal pocket **FD** fertilization duct **GA** glandular appendage **LL** lateral lobes **LS** lobal septum **MS** membranous duct **SP** spermophore **SS** slit sensillum **ST** subtegulum **TE** tegulum **vRTA** ventral branch of retrolateral tibial apophysis. Scale bars: 2.0 mm (**A–C**); 0.5 mm (**D, E**).

**Figure 12.**
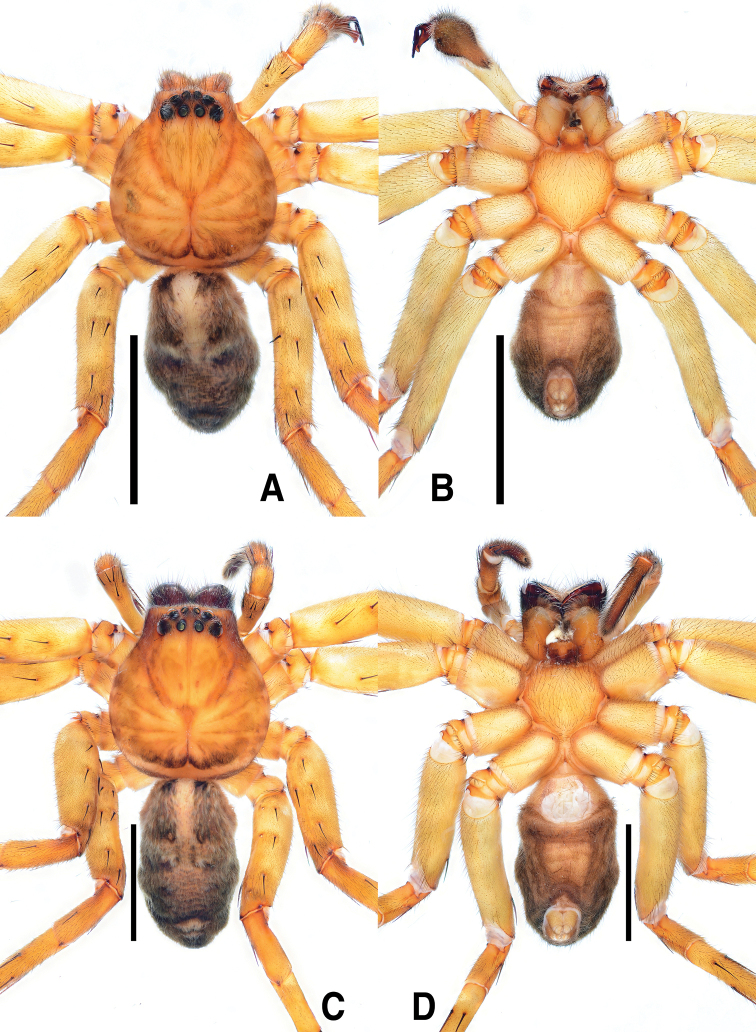
*Sinopodapantherina* sp. nov., habitus in ethanol **A, B** male holotype (**A** dorsal **B** ventral) **C, D** female paratype (**C** dorsal **D** ventral). Scale bars: 5.0 mm.

**Figure 13.**
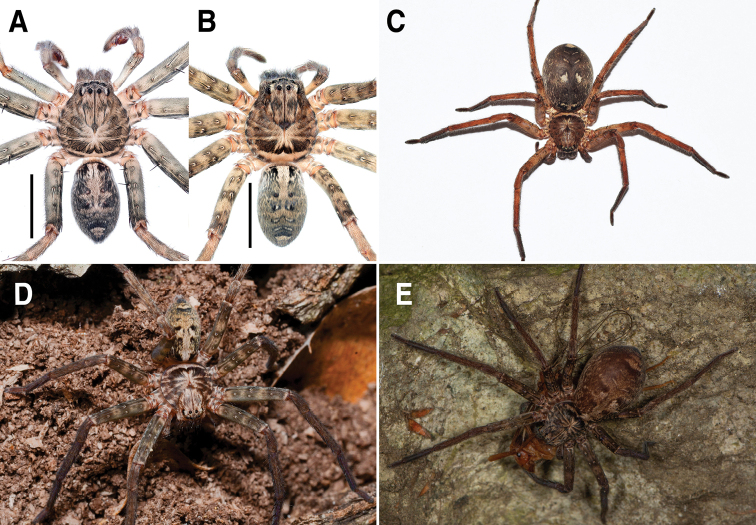
Live statements of *Sinopoda* spp. **A, B***Sinopodabogil* sp. nov. (**A** male, dorsal view **B** female, dorsal view) **C***Sinopodabigibba* sp. nov., dorsal view of paratype female from Taean-gun **D, E***Sinopodapantherina* sp. nov., from Geoje-si (**D** dorsal view of juvenile female **E** dorsal view of adult female). Scale bars: 5.0 mm (**A, B**).

Figs 10 , 11 , 12 , 13

##### Type material.

***Holotype*** ♂ **Republic Of Korea**: Gyeongsangnam-do, Geoje-si, Gohyeon-dong, rock piles above leaf litter, walls; 34°52.20'N, 128°36.73'E; ca. 454 m; 14 Jul. 2016; J. Chae leg. ***Paratype*** 1 ♀ same data as holotype.

##### Etymology.

The specific epithet *pantherina* is derived from the Latin adjective *pantherinus*, -*a*, -*um*, meaning leopard-like, originating from the coloration pattern of live specimens (Fig. 13D, E).

##### Diagnosis.

This species can be distinguished from other congeners by the combination of following characteristics: Male―distal portion of cymbium distinctly bent ventrally; embolus with membranous flange extended prolaterally; embolic apophysis distally truncated with membranous flange slightly extended ventrally; spermophore strongly curved; vRTA slightly curved inwardly and distally blunt in ventral view, thumb-shaped in retrolateral view. Female―epigyne with slightly elongated sclerotized epigynal bulges; lateral lobes with indistinct median furrow, posteromedially with deep and wide indentation; anterolateral margin of lateral lobes almost linear, posterior margin without humps; lobal septum narrow and triangular, without indentation; glandular appendages nearly linear, slightly longer than posterior part of vulva.

##### Description.

**Male (*holotype*) Measurements**: Total Length: 11.20, PL: 5.32, PW: 5.47, OL: 5.88, OW: 3.66, AW: 2.87. ***Eyes***: AME: 0.25, ALE: 0.37, PME: 0.30, PLE: 0.42, AME–AME: 0.17, AME–ALE: 0.09, PME–PME: 0.25, PME–PLE: 0.37, AME–PME: 0.40, ALE–PLE: 0.37, clypeus AME: 1.11, clypeus ALE: 1.10. ***Palp***: 5.76 (1.74, 1.08, 0.83, 2.11). ***Legs***: I 30.84 (7.58, 2.24, 8.71, 9.19, 3.12), II 35.02 (9.33, 2.63, 9.79, 10.10, 3.17), III 27.11 (7.23, 2.85, 6.85, 7.30, 2.88), IV 29.35 (7.59, 2.59, 7.50, 8.95, 2.72). Leg formula: II-IV-I-III. **Spination: *Palp***: 131, 101, 2111, 1000. ***Legs***: Fe I–II 323, III 302, IV 321, Pa I–III 101, IV 001, Ti I 1328, II 1318, III 2126, IV 2226, Mt I 1024, II 1216, III 1016, IV 2026. ***Chelicerae***: furrow with three anterior and four posterior teeth.

***Palp***: As per diagnosis (Figs 10A–C, 11A–C). Distal portion of cymbium distinctly bent ventrally. Embolus slender, arising from tegulum at 6:30–7-o’clock-position, shorter than embolic apophysis, distally curved with membranous flange extended prolaterally. Embolic apophysis wider than embolus, curved perpendicularly, distally truncated. Conductor arising from tegulum at 1:00–1:30-o’ clock-position. Tegulum slightly covered proximal portion of embolus. dRTA longer than vRTA, curved nearly perpendicularly and distally tapered. vRTA distinctly wider than dRTA in retrolateral view.

**Coloration in ethanol.** (Fig. 12A, B): ***Prosoma***: Carapace yellowish brown, covered with dark brown hairs making radial pattern, posterior margin with pale yellow horizontal band. Cervical groove and median groove reddish brown. Sternum pale yellow. ***Opisthosoma***: dorsally covered with dark grey hairs, anterior portion with pair of irregular black spots laterally and longitudinal ivory stripe medially, ventrally brown medially, and laterally dark brown. ***Chelicerae***: reddish brown with brown stripes. ***Palp and legs***: yellowish brown.

**Female (*paratype*) Measurements**: Total length: 15.18, PL: 7.64, PW: 6.72, OL: 7.54, OW: 4.20, AW: 3.98. ***Eyes***: AME: 0.27, ALE: 0.49, PME: 0.30, PLE: 0.47, AME–AME: 0.27, AME–ALE: 0.13, PME–PME: 0.45, PME–PLE: 0.53, AME–PME: 0.43, ALE–PLE: 0.54, clypeus AME: 0.47, clypeus ALE: 0.43. ***Palp***: 9.52 (3.11, 1.37, 2.04, 3.00). ***Legs***: I 26.38 (7.56, 3.13, 7.04, 6.61, 2.04), II 26.93 (7.96, 3.47, 7.56, 5.94, 2.00), III 24.40 (7.21, 2.89, 6.29, 5.71, 2.30), IV 26.92 (7.42, 3.02, 6.84, 7.21, 2.43). Leg formula: II-IV-I-III. **Spination: *Palp***: 131, 101, 2121, 1014. ***Legs***: Fe I–II 323, III 332/322, IV 331, Pa I 001, II–III 101, IV 101/001, Ti I 1018, II 1118, III–IV 2126, Mt I 0004, II 1014, III–IV 2026. ***Chelicerae***: furrow with three anterior and four posterior teeth.

***Copulatory organ***: As per diagnosis (Figs 10D, E, 11D, E). Epigynal field wider than long, with slit sensilla, anteromedially with weakly elongated sclerotized epigynal bulges. Epigynal pockets running from laterally to anteromedially. Internal duct system long as wide, anteriorly slightly bulged, posterior part slightly shorter than anterior part. Glandular appendage distally rounded, pointing posterolaterally. Median part of vulva as long as posterior part. Fertilization ducts curved and pointing posterolaterally.

**Coloration in ethanol.** (Fig. 12C, D): Generally same as male, but slightly darker.

**Coloration in live specimen.** (Fig. 13D, E): ***Prosoma***: Carapace covered with pale brown hairs, cephalic area with pair of dark brown marks and dark brown median longitudinal line, thoracic area with many dark brown marks, making radial pattern. ***Opisthosoma***: reddish brown, anteromedially with pale brown longitudinal mark, medially with two pairs of black spots on muscle sigillae, posterior muscle sigillae with pair of small ivory marks anteriorly, posteriorly with two pairs of ivory chevrons, one large triangular mark. ***Palp and legs***: reddish brown, distally darker, spines with ivory dots, and dark brown ring patterns.

**Figure 14.**
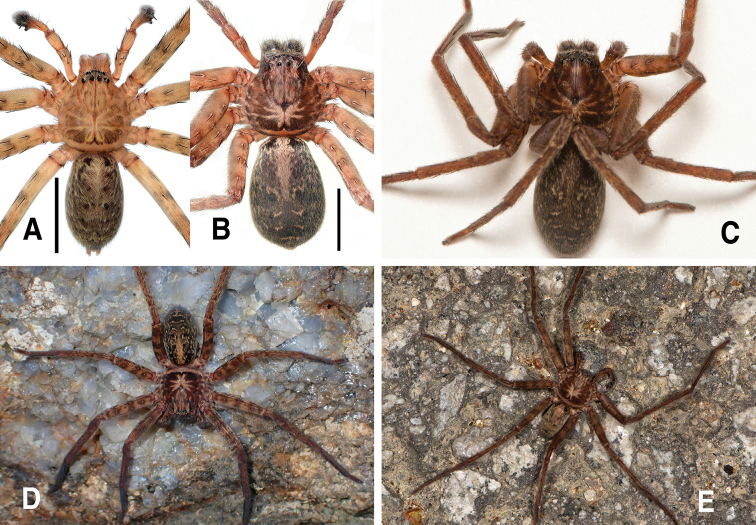
Live statements of *Sinopodajirisanensis* Kim & Chae, 2013 **A, B** specimens from Mt. Jirisan (**A** male, dorsal view **B** female, dorsal view) **C** female from Mt. Odaesan **D** female from Mt. Sobaeksan **E** male from Mt. Gakhwasan. Scale bars: 5.0 mm (**A, B**).

##### Distribution.

**Figure 15.**
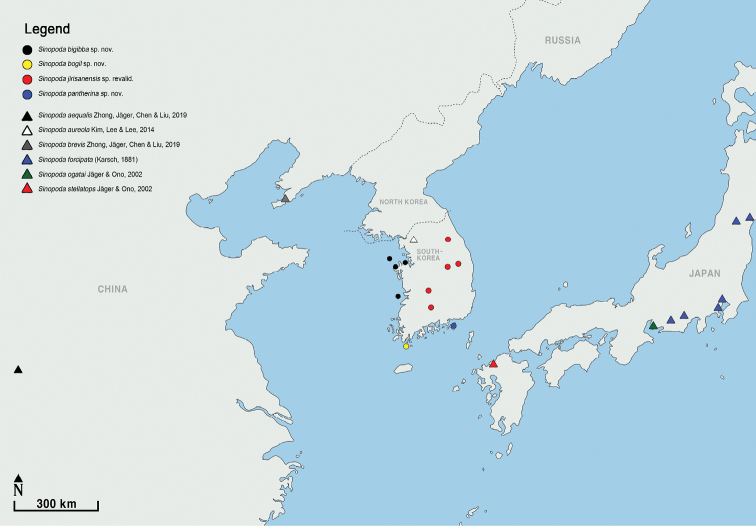
Distribution map of new and revalidated *Sinopoda* species and its comparable species in Korea, Japan, and China. A circle indicates the new species and reinstated species; a triangle indicates the other recorded species.

Republic of Korea (known only from the type locality) (Fig. 15).

##### Remarks.

The male of *Sinopodapantherina* sp. nov. is similar to *Sinopodabigibba* sp. nov. (Figs 1A–C, 2A–C) in having broadened membranous structure and similar RTA but can be distinguished from the latter by: 1) embolic apophysis distally blunt and truncated (distally tapered in *S.bigibba*), 2) spermophore slightly curved in ventral view (distinctly curved in *S.bigibba*), and 3) vRTA 1.5 × wider than dRTA in retrolateral view (~ 3 × wider than dRTA in retrolateral view in *S.bigibba*).

Female of *Sinopodapantherina* sp. nov. is similar to *Sinopodaaureola* Kim, Lee & Lee, 2014 (Kim et al. 2014: 282, figs 3E, F, 4E, F) in having epigynal bulges and glandular appendages directed posterolaterally, however this species can be distinguished from *S.aureola* by: 1) lobal septum without median indentation (with medial longitudinal indentation in *S.aureola*), 2) glandular appendages linear, slightly longer than posterior part of internal ducts (posteriorly curved, distinctly longer than posterior part of internal ducts in *S.aureola*), and 3) reddish brown coloration in live habitus (yellowish brown in *S.aureola*).

## Supplementary Material

XML Treatment for Sinopoda

XML Treatment for Sinopoda bigibba

XML Treatment for Sinopoda bogil

XML Treatment for Sinopoda jirisanensis

XML Treatment for Sinopoda pantherina
